# Identification and characterization of meat allergens for improved diagnosis of meat allergy

**DOI:** 10.1186/2045-7022-3-S3-P174

**Published:** 2013-07-25

**Authors:** C Klug, M Focke, W Hemmer, H Wank, I Swoboda

**Affiliations:** 1 Department Applied Life Sciences, Universitiy of Applied Sciences FH Campus Vienna, Vienna, Austria; 2 Department of Pathophysiology and Allergy Research, Medical University of Vienna, Vienna, Austria; 3 Floridsdorf Allergy Center (FAZ), Vienna, Austria

## Background

In Western European countries meat represents a mainstay of the diet. However, meat can also induce severe allergic reactions. About 0.5 to 8% of all food allergies are caused by various meat sources. Besides the observation that meat allergic patients are either sensitized to white meat (e.g., chicken, turkey) or to red meat (e.g., beef, pork, lamb), there is limited knowledge on allergic reactions caused by meat and only a few meat allergens have so far been identified. Diagnosis of meat allergy is currently still based on poorly standardized extracts with unsatisfactory sensitivity and specificity. With the final goal to develop tools for improved meat allergy diagnosis, we aimed to thoroughly characterize IgE reactive proteins of meat sources regarded as the most common causes of meat allergy in Central Europe, namely chicken, beef and pork.

## Methods

We prepared aqueous protein extracts from muscle tissue of chicken, beef and pork, separated them by SDS-PAGE and blotted them onto nitrocellulose membranes and incubated them either with sera from patients sensitized to chicken or with sera from patients sensitized to red meat. Protein bands recognized by several patients were excised from the gels, subjected to trypsin digestion and analysed by mass spectrometry (LC-ESI MS/MS).

## Results

Comparison of the IgE reactivity profiles allowed for each meat source the identification of proteins, which were recognized by the majority of the patients sensitized to the meat source, indicating the presence of major meat allergens. Subsequent mass spectrometry of these IgE-reactive bands enabled us to identify several potential meat allergens, among them also serum albumin, a protein that has already previously been suggested to be involved in allergic reactions against read meat.

## Conclusion

Based on the sequences of the putative allergens we will design specific primers for cDNA cloning and generation of recombinant allergens for improved, component-resolved meat allergy diagnosis.

## Disclosure of interest

None declared.

